# Effects of ambient temperature and relative humidity on preterm birth during early pregnancy and before parturition in China from 2010 to 2018: a population-based large-sample cohort study

**DOI:** 10.3389/fpubh.2023.1101283

**Published:** 2023-06-20

**Authors:** Yu Wu, Jie Yuan, Yanling Yuan, Cai Kong, Wenzhan Jing, Jue Liu, Hanfeng Ye, Min Liu

**Affiliations:** ^1^Department of Epidemiology and Biostatics, School of Public Health, Peking University, Beijing, China; ^2^Yunnan Population and Family Planning Research Institute, Yunnan, China

**Keywords:** temperature, humidity, preterm birth, China, cohort

## Abstract

**Background:**

The progression of global warming and increase in instances of extreme weather have received considerable attention. We conducted a cohort study on women of childbearing age in Yunnan Province, examined the association between ambient temperature and humidity on preterm birth and evaluated the effects of extreme weather during early pregnancy and before parturition on preterm birth.

**Methods:**

We conducted a population-based cohort study on women of childbearing age 18–49 years who participated in National Free Preconception Health Examination Project (NFPHEP) in Yunnan Province from January 1, 2010, to December 31, 2018. Meteorological data, namely daily average temperature (°C) and daily average relative humidity (%), were obtained from China National Meteorological Information Center. Four exposure windows were explored: 1 week of pregnancy, 4 weeks of pregnancy, 4 weeks before delivery, and 1 week before delivery. We used a Cox proportional hazards model and adjusted the potential risk factors for preterm birth to obtain the effects of exposure to temperature and humidity on preterm birth among the stages of pregnancy.

**Results:**

At 1 week of pregnancy and at 4 weeks of pregnancy, the association between temperature and preterm birth was U-shaped. The correlation between relative humidity and the risk of preterm birth was n-type at 1 week of pregnancy. The correlation between preterm birth and temperature and relative humidity at 4 weeks before delivery and at 1 week before delivery is J-shaped. Low temperature and low humidity were protective factors against preterm birth, whereas high temperature and high humidity were risk factors for preterm birth.

The effects of high temperature and extremely high temperature were the strongest at 4 weeks before delivery, with HRs of 1.417 (95% CI: 1.362–1.474) and 1.627 (95% CI: 1.537–1.722), respectively. The effects of extremely low humidity and low humidity were strongest at 1 week before delivery, with HRs of 0.681 (95% CI: 0.609–0.761) and 0.696 (95% CI: 0.627–0.771), respectively.

**Conclusion:**

Temperature and relative humidity affect preterm birth differently for each pregnancy stage. The effects of meteorological factors on pregnancy outcomes such as premature birth should not be ignored.

## 1. Introduction

The progression of global warming and increase in instances of extreme weather have received considerable attention, and numerous studies have investigated diseases that may be related to environmental factors (1). Seasonal weather changes strongly affect human health. Studies have reported that exposure to low and high temperatures was associated with mortality and morbidity related to the cardiopulmonary system, infections, and mental health (2–4). Studies have also indicated that exposure to low and high temperatures could restrict the circulation system and induce cytokine release, which were hypothesized to potentially affect pregnancy (5). Pregnancy increases women’s vulnerability to environmental hazards and factors (6). Pregnancy can also increase social vulnerability, especially in low- and middle-income countries, where women often perform household chores and work long hours outdoors to avoid forgoing pay during pregnancy (7). Exposure to environmental factors during pregnancy and their potential association with adverse pregnancy outcomes such as preterm birth are major public health concerns.

Globally, preterm birth complications are the second leading cause of death among children under 5 years of age, after pneumonia (8). Approximately 1 million children under 5 years of age die each year because of preterm birth complications. Approximately 944,000 neonates died from preterm birth every year, accounting for 35.3% of all neonatal deaths (9). In almost all countries with reliable data, preterm birth rates are increasing. More than 60% of preterm births occur in Africa and South Asia, but preterm birth is a global problem. In low-income countries, the prevalence of preterm birth is 12% on average, and in high-income countries, the prevalence is 9% (9). With a large population and high number of births, China has the second most preterm births in the world (9). Therefore, improving the problem of preterm births is critical to reducing neonatal and childhood mortality and morbidity.

China possesses a large territory comprising a variety of natural environments. Therefore, the association between premature birth and temperature and humidity may differ by region. Most national studies have analyzed meteorological factors by province, and the results have been inaccurate because of the high granularity. Our study focused on Yunnan Province, China, which includes 16 cities (Kunming, Qujing, Yuxi, Baoshan, Zhaotong, Lijiang, Puer, Lincang, Chuxiong Autonomous Prefecture, Honghe Hani and Yi Autonomous Prefecture, Wenshan Zhuang and Miao Autonomous Prefecture, Xishuangbanna Dai Autonomous Prefecture, Dali Bai Autonomous Prefecture, Dehong Dai and Jingpo Autonomous Prefecture, Nujiang Lisu Autonomous Prefecture, and Diqing Tibetan Autonomous Prefecture), and we analyzed the basic characteristics of temperature and humidity by city. The low granularity ensured that the association between preterm birth and temperature and humidity was accurate.

Yunnan is at a subtropical northern latitude and has a climate typical of such a region. The average annual temperature is above 15°C, and the average annual precipitation is 750–1750 mm. Precipitation is unevenly distributed throughout the year, with distinct dry and rainy seasons. In addition, Yunnan has the most ethnic groups in China but is less economically developed than the rest of the country; the province exhibits regional economic disparity, and medical and health resources are also unevenly distributed. Therefore, targeted assistance and interventions must be implemented to improve its health services and poverty relief initiatives (10).

Studies have indicated that high temperatures were associated with a high risk of preterm birth (11, 12). However, this association varies by country, population, and pregnancy stage. Studies have mainly investigated the association between temperature and preterm birth, and few studies have investigated the joint effect of temperature and humidity on preterm birth. To explore the effects of environmental factors during pregnancy on preterm birth, we conducted a cohort study on women of childbearing age in Yunnan Province, examined the association between ambient temperature and humidity on preterm birth and evaluated the effects of extreme weather during early pregnancy and before parturition on preterm birth.

## 2. Methods

We conducted a population-based cohort study on women who attempted to become pregnant and participated in the National Free Preconception Health Examination Project (NFPHEP) from January 1, 2010, to December 31, 2018, in 16 cities in Yunnan Province. The NFPHEP was launched by the Chinese National Health and Family Planning Commission in 2010 to provide free health examinations and other services before conception for couples who plan to become pregnant within 6 months as well as follow-up during the first trimester of pregnancy and after delivery. This study was approved by the Institutional Review Board of the Chinese Association of Maternal and Child Health Studies (AMCHS-2014-6). All participants provided written informed consent before enrollment (13).

Trained and qualified local health workers used a standardized questionnaire to collect baseline information from the women who participated in the NFPHEP, namely age, education level, history of childbirth, and adverse pregnancy outcomes. The health workers measured the participants’ height, weight, and concentration of hemoglobin and calculated their body mass index (BMI). For the diagnostic criteria for anemia, this study referenced the diagnostic threshold recommended by the World Health Organization [lower than 120 g/L for nonpregnant women (14)]. Maternal and child health specialists interviewed the participants 3 months after conception and noted the participants’ last menstrual period and any habits of smoking or drinking alcohol during pregnancy. The health workers followed up with all participants 1 month after delivery and noted their delivery method, pregnancy outcome, delivery date, gestational age, and information regarding the newborn (sex and singleton or multiple birth). The study ended when the participants had a preterm birth or other outcome or when the observation period ended (December 31, 2018).

### 2.1. Outcomes

Gestational age was the first day of the last menstruation until the end of the process, approximately 280 days (40 weeks). The primary outcome was preterm birth, which was defined as a delivery from 28 weeks to less than 37 weeks of gestation (9). The preterm birth rate was the proportion of premature births among the total number of all singleton livebirths.

### 2.2. Exposure

Meteorological data from 16 cities in Yunnan Province, namely daily average temperature (°C) and daily average relative humidity (%) between 2010 to 2018, were obtained from China National Meteorological Information Center. Because the etiological threshold for the effects of temperature and relative humidity exposure on risk of preterm birth is unclear, several exposure windows were explored.

The windows of exposure were 1 week of pregnancy, 4 weeks of pregnancy, 4 weeks before delivery, and 1 week before delivery. Term births were censored at the end of the 36th week of gestation because the risk of preterm birth was eliminated (15). For full-term births, 1 and 4 weeks before delivery were considered weeks 33–36 after pregnancy and the 36th week after pregnancy, respectively.

The daily exposure temperature and relative humidity were matched to each pregnant woman according to the date of conception, and the average exposure in each window was calculated to identify the association between premature delivery and temperature and humidity. Extremely low temperature and humidity were the first percentile of temperature and humidity distribution. Low temperature and humidity were the fifth percentile of the temperature and humidity distribution. High temperature and humidity were the 95th percentile of the temperature and humidity distribution. Extremely high temperature and humidity were the 99th percentile of the temperature and humidity distribution (16).

### 2.3. Covariates

Covariates related to preterm birth, namely age (≤20, 21–25, 26–30, 31–35, and > 35 years of age), education (elementary school and below, middle school, high school, and college or higher), history of delivery (yes or no), history of adverse pregnancy outcomes (yes or no), BMI (<18.5, 18.5–23.9, 24.0–27.9, and ≥ 28.0 kg/m^2^), anemia (yes or no), habit of smoking during pregnancy (yes or no), habit of drinking alcohol during pregnancy (yes or no), and fetal sex (male or female), were adjusted.

### 2.4. Statistical analysis

Length of exposure may differ by gestational age. To account for differences in birth outcome and exposure time, we used a Cox proportional hazards model with gestational age as the time axis and preterm birth as the outcome to study the effects of exposure to temperature and humidity on preterm birth among the stages of pregnancy. We first modeled time-varying weekly temperatures during each time window as a cubic spline with three degrees of freedom (df) to create a nonlinear relationship with preterm birth and then incorporated the basis function into the Cox proportional hazards model to form the basic model (17). We then incorporated other covariates into the model. Similar to temperature, relative humidity was used as a time-dependent variable during each exposure window by using a spline with three df. The formula for the Cox proportional hazards model is as follows:


$$\begin{array}{c} h\left(GA,PTB\right)=h_{0}\left(GA\right)\exp\left(cb\left(MM_{i},df=3\right)\right)+\beta_{1}MAG+\beta_{2}CL\\ +\beta_{3}PD+\beta_{4}AP+\beta_{5}BMI+\beta_{6}AN+\beta_{7}AL\\ +\beta_{8}SK+\beta_{9}GD, \end{array}$$

where *GA* is the time variable, gestational age, *PTB* is whether the child was born prematurely, *cb()* represents the basis function, *MMi* represents the average value of the meteorological variables in the *i*th window of exposure, *df* is the degree of freedom, *β* is a coefficient, *MAG* is the age of the mother, *CL* is the maternal education level, *PD* is the history of delivery, *AP* is the history of adverse pregnancy outcomes, *BMI* is the body mass index, *AN* is anemia, *AL* is a habit of smoking during pregnancy, *SK* is a habit of drinking during pregnancy, and *GD* is the fetal sex.

Based on the results of the Cox model, we plotted the risk exposure–response curves for temperature and relative humidity during pregnancy with respect to preterm birth to represent the temperature and relative humidity distribution. In addition, we calculated specific hazard ratios (HRs) and 95% confidence intervals (CIs) relative to the median temperature for extreme temperature and humidity. All statistical analyzes were performed using R (version 4.0.2). The “survival” and “smoothHR” packages were used to create a Cox proportional risk regression model with a significance level of 0.05 on both sides.

## 3. Results

This study recruited 214,695 participants, of whom 2,684 had missing delivery time or address data, 6 were younger than 13 years of age or older than 50 years of age, 1,132 had multiple births, 589 had stillbirths, 3,329 had a gestational age of less than 20 weeks or more than 44 weeks, and 1,184 had an abortion or induced labor—these participants were excluded, and 205,771 participants remained (Supplementary Figure S1). Supplementary Table S1 presents the basic characteristics of the participants.

### 3.1. Meteorological data and preterm birth by cities and exposure window

The lowest mean temperature was observed in Diqing Tibetan Autonomous Prefecture (5.87–6.98°C), and the highest was observed in Xishuangbanna Dai Autonomous Prefecture (21.6–22.3°C). The lowest average relative humidity was observed in Lijiang (58.8–61.6%), and the highest was observed in Xishuangbanna Dai Autonomous Prefecture (82.4–83.1%). Of the 205,771 participants, 18,014 had preterm births, accounting for 8.75% of the participants. Qujing had the lowest preterm birth rate: 4.63%. Diqing Tibetan Autonomous Prefecture had the highest preterm birth rate: 19.08% (Table 1).

**Table 1:** Meteorological data and preterm births in 16 cities of Yunnan province in different exposure windows.

City	Number of pregnant women (*n*)	Exposure windows								Preterm birth *n* (%)
		1 week of pregnancy		4 weeks of pregnancy		1 week before delivery		4 weeks before delivery		
		Average temperature (°C)	Average relative humidity (%)	Average temperature (°C)	Average relative humidity (%)	Average temperature (°C)	Average relative humidity (%)	Average temperature (°C)	Average relative humidity (%)	
Kunming	11,991	15.80	71.30	15.80	71.40	15.60	71.40	15.60	71.50	1,192 (9.94)
Qujing	24,025	14.00	69.40	14.10	69.90	12.40	69.90	12.30	69.70	1,112 (4.63)
Yuxi	22,732	16.70	70.70	16.60	70.60	16.80	71.70	16.80	71.80	1967 (8.65)
Baoshan	4,826	18.00	67.30	18.00	66.90	17.50	64.60	17.60	64.80	510 (10.57)
Zhaotong	9,297	12.80	73.80	12.70	73.90	12.30	74.30	12.30	74.10	969 (10.42)
Lijiang	8,609	14.50	61.60	14.30	61.00	13.90	58.80	13.90	59.90	532 (6.18)
Puer	8,504	19.90	76.30	19.90	76.40	19.40	76.10	19.40	76.20	847 (9.96)
Lincang	11,832	18.10	68.80	18.10	68.40	18.10	69.90	18.10	70.40	854 (7.22)
Chuxiong Yi Autonomous Prefecture	11,231	19.20	73.40	19.30	73.10	19.10	74.70	19.00	74.80	1,458 (12.98)
Honghe Hani and Yi Autonomous Prefecture	42,694	15.20	74.60	15.30	74.50	15.80	77.20	15.70	77.00	2,300 (5.39)
Wenshan Zhuang and Miao Autonomous Prefecture	15,073	17.00	77.90	17.00	77.90	18.40	79.80	18.30	79.80	2,345 (15.56)
Xishuangbanna Dai Autonomous Prefecture	4,654	22.30	82.50	22.20	82.40	21.60	83.10	21.60	83.10	734 (15.77)
Dali Bai Autonomous Prefecture	17,221	15.40	64.90	15.50	64.60	15.70	67.50	15.60	67.40	1,441 (8.37)
Dehong Dai and Jingpo Autonomous Prefecture	9,445	15.70	78.90	15.70	78.70	15.40	78.80	15.40	79.10	1,172 (12.41)
Nujiang Lisu Autonomous Prefecture	1928	20.50	63.40	20.70	63.60	20.50	66.70	20.30	66.60	255 (13.23)
Diqing Tibetan Autonomous Prefecture	1709	6.84	64.10	6.98	63.70	5.91	63.70	5.87	64.00	326 (19.08)
Total	205,771	16.08	71.91	16.09	71.82	15.99	73.04	16.03	73.05	18,014 (8.75)

### 3.2. Effects of temperature and relative humidity on preterm birth by exposure window

Supplementary Table S2 displays the distribution of temperature and relative humidity by exposure window. The mean daily temperatures 1 week of pregnancy, 4 weeks of pregnancy, 4 weeks before delivery, and 1 week before delivery were 16.08, 16.09, 15.99, and 16.03°C, respectively. The mean daily relative humidity 1 week of pregnancy, 4 weeks of pregnancy, 4 weeks before delivery, and 1 week before delivery was 71.91, 71.82, 73.05, and 73.04%, respectively. The median daily mean temperature (P50) 1 week of pregnancy, 4 weeks of pregnancy, 4 weeks before delivery, and 1 week before delivery was 16.97, 16.94, 16.89, and 16.95°C, respectively. The median daily relative humidity (P50) 1 week of pregnancy, 4 weeks of pregnancy, 4 weeks before delivery, and 1 week before delivery was 75.5, 74.75, 81.48, and 82.14%, respectively (Table 2).

**Table 2:** Distribution of temperature and relative humidity by exposure window.

Meteorological factors		1 week of pregnancy	4 weeks of pregnancy	4 weeks before delivery	1 week before delivery
Temperature (°C)	Average	16.08	16.09	15.99	16.03
	P1 (Extreme cold)	2.64	4.29	1.99	3.52
	P5 (Cold)	6.67	7.43	6.29	7.08
	P25	12.14	12.25	12.14	12.27
	P50 (Median)	16.97	16.94	16.89	16.95
	P75	20.27	20.14	20.24	20.16
	P95 (Hot)	23.26	22.92	23.09	22.79
	P99 (Extreme hot)	25.26	25.00	25.04	24.84
Relative humidity (%)	Average	71.91	71.82	73.05	73.04
	P1(Extreme dry)	35.86	40.93	51.32	48.71
	P5(Dry)	46.39	49.46	66.19	65.84
	P25	64.36	64.84	76.36	76.57
	P50	75.50	74.75	81.48	82.14
	P75	81.57	80.88	85.24	87.50
	P95(Wet)	87.18	84.92	88.69	91.11
	P99(Extreme wet)	91.00	88.82	43.15	38.43

**Figure 1:**
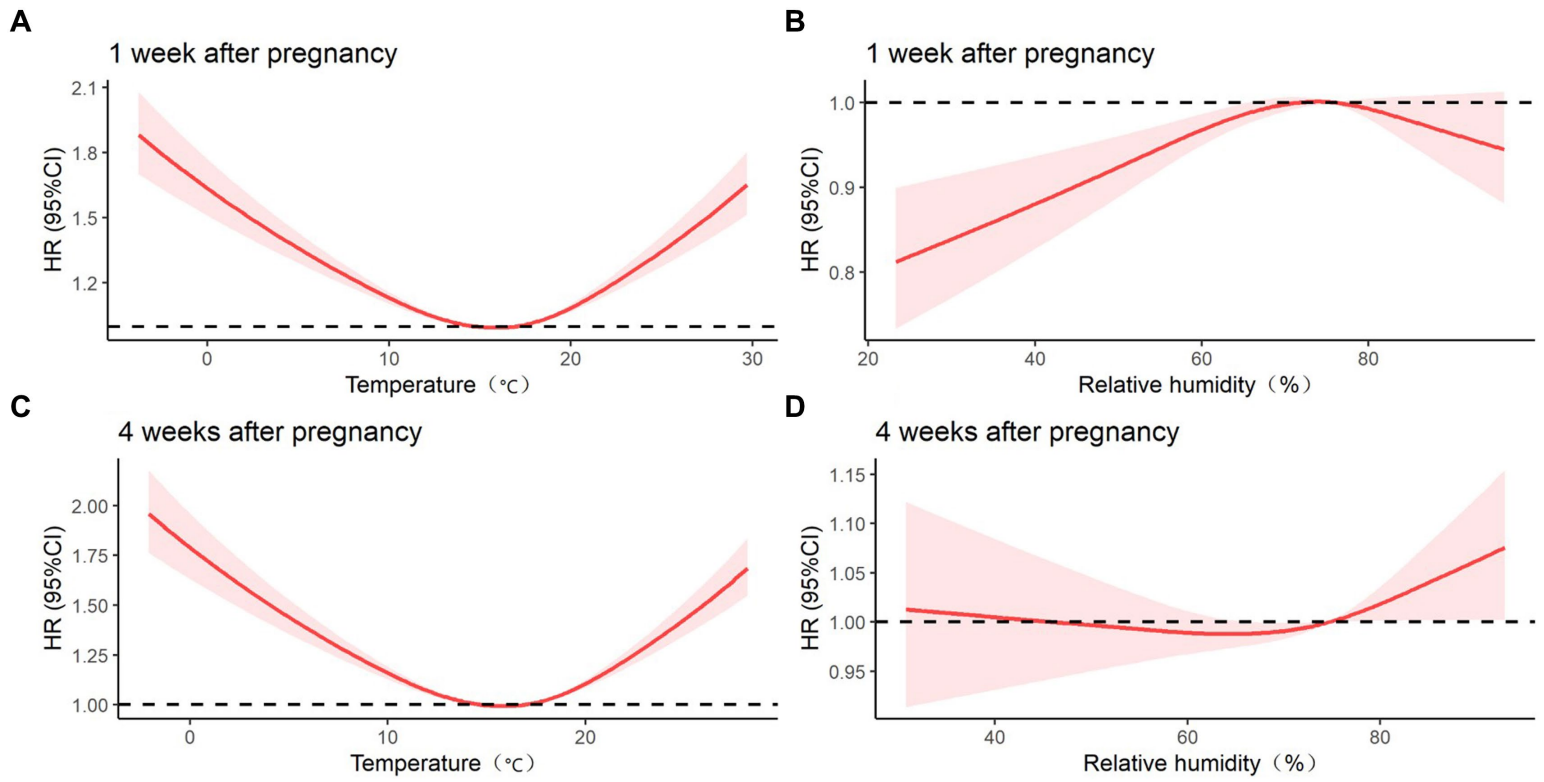
Effect of temperature and relative humidity on preterm birth at 1 week of pregnancy and 4 weeks of pregnancy after adjusting for covariates such as age, education, history of delivery, history of adverse pregnancy outcomes, BMI, anemia, smoking during pregnancy, drinking during pregnancy, and fetal sex. **(A)** COX regression model of temperature at 1 week of pregnancy and preterm birth; **(B)** COX regression model of relative humidity at 1 week of pregnancy and preterm birth; **(C)** COX regression model of temperature at 4 weeks of pregnancy and preterm birth; **(D)** COX regression model of relative humidity at 4 weeks of pregnancy and preterm birth.

Figure 1 presents the association between the risk of preterm birth and temperature and relative humidity at 1 week of pregnancy and at 4 weeks of pregnancy. At these times, the association between temperature and preterm birth was U-shaped, and the risk of preterm birth was lowest at 13–18°C. The correlation between relative humidity and the risk of preterm birth was n-type at 1 week of pregnancy, and the risk of preterm birth was highest when relative humidity was approximately 70–80%. No association between relative humidity at 4 weeks of pregnancy and preterm birth was observed.

**Figure 2:**
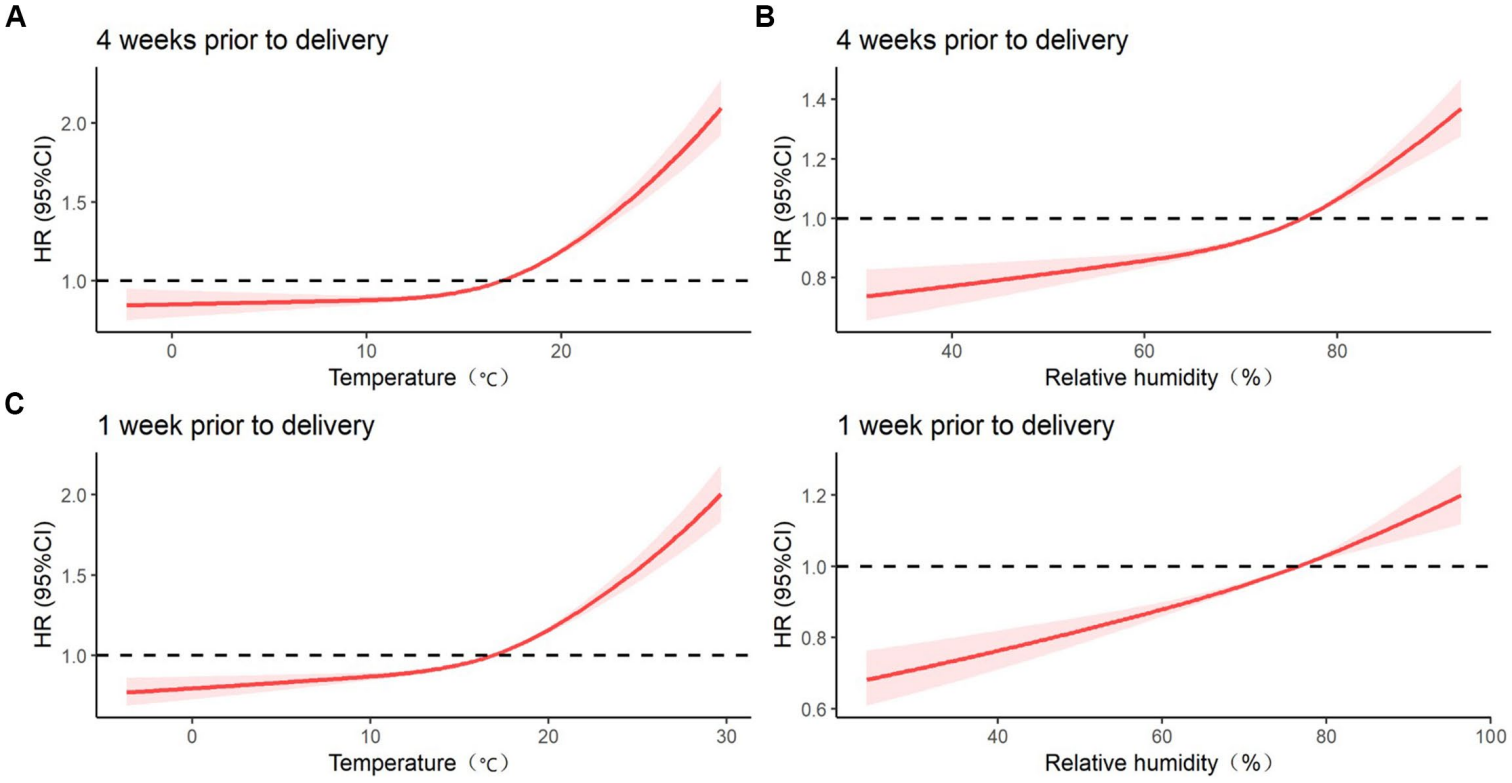
Effect of temperature and relative humidity on preterm birth during 4 weeks before delivery and 1 week before delivery after adjusting for covariates such as age, education, history of delivery, history of adverse pregnancy outcomes, BMI, anemia, smoking during pregnancy, drinking during pregnancy, and fetal sex. **(A)** COX regression model between temperature 4 weeks before delivery and preterm birth; **(B)** COX regression model of relative humidity 4 weeks before delivery and preterm birth; **(C)** COX regression model between temperature 1 week before delivery and preterm birth; **(D)** COX regression model of relative humidity 1 week before delivery and preterm birth.

Figure 2 presents the association between temperature and relative humidity during the 4 weeks before delivery and at 1 week before delivery for a preterm birth. The correlation between preterm birth and temperature and relative humidity at 4 weeks before delivery and at 1 week before delivery is J-shaped. Low temperature and low humidity were protective factors against preterm birth, whereas high temperature and high humidity were risk factors for preterm birth. An increased risk of preterm birth was significantly associated with high temperature during all four exposure windows (*p* < 0.05).

### 3.3. Effects of extreme weather on preterm birth by exposure window

**Figure 3:**
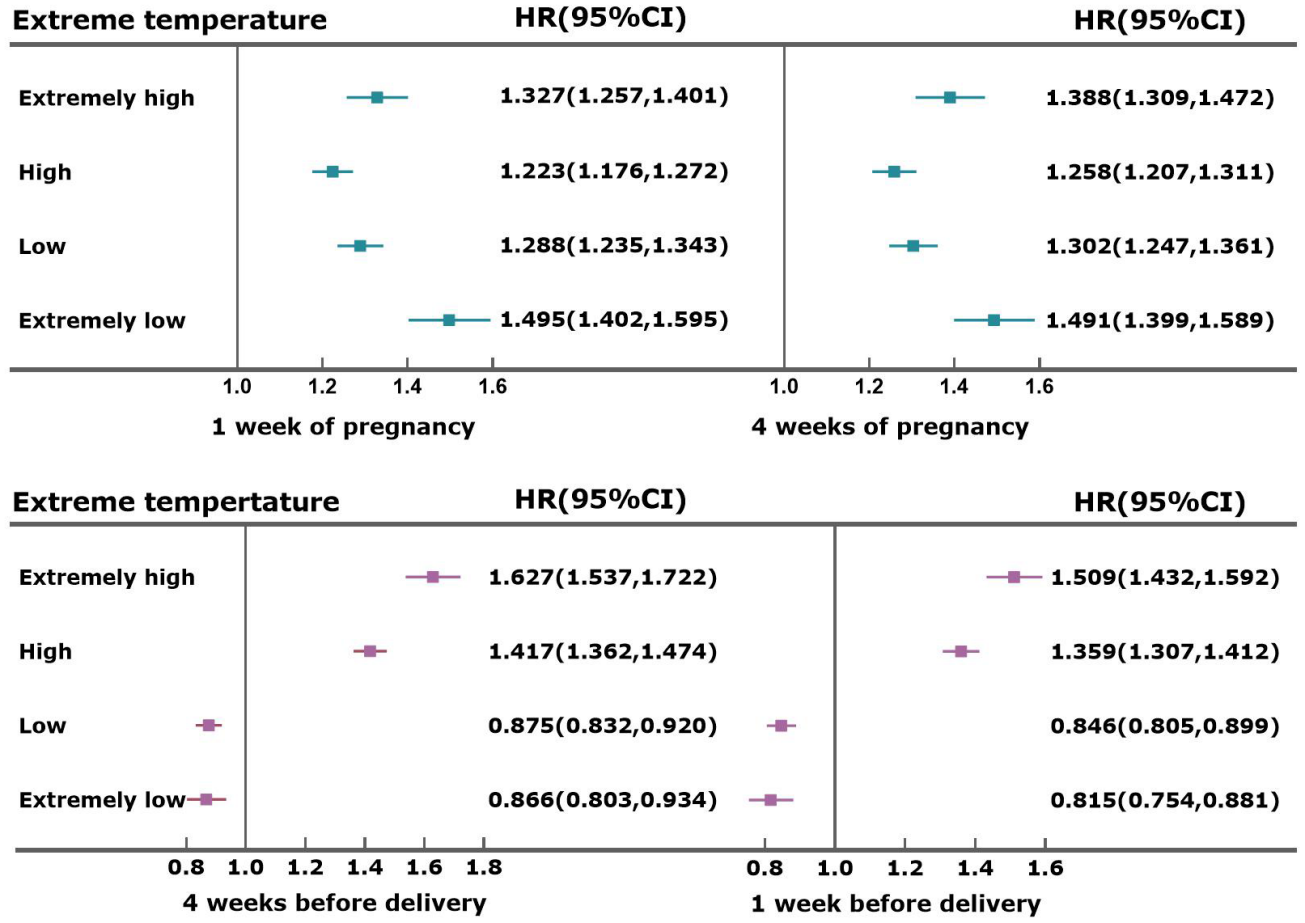
Effect of extreme temperature on preterm birth by exposure window.

Figure 3 presents the results of the Cox proportional risk model for the effect of exposure to extreme temperature on preterm birth by exposure window. The results indicated that extremely low temperature (P1) and low temperature (P5) at 1 week of pregnancy and at 4 weeks of pregnancy were both risk factors for preterm birth and that extremely low temperature (P1) and low temperature (P5) at 4 weeks before delivery and at 1 week before delivery were protective factors against preterm birth. High temperature (P95) and extremely high temperature (P99) were both risk factors for preterm labor in the four exposure windows, and the effects of high temperature (P95) and extremely high temperature (P99) were the strongest at 4 weeks before delivery, with HRs of 1.417 (95% CI: 1.362–1.474) and 1.627 (95% CI: 1.537–1.722), respectively.

Supplementary Figure S2 presents the results of the Cox proportional risk model for the effects of exposure to extreme humidity on preterm birth for each exposure window. Extremely low humidity (P1) and low humidity (P5) at 1 week of pregnancy, 4 weeks before delivery, and at 1 week before delivery were protective factors against preterm birth, and the effects were strongest at 1 week before delivery, with HRs of 0.681 (95% CI: 0.609–0.761) and 0.696 (95% CI: 0.627–0.771), respectively. High humidity (P95) and extremely high humidity (P99) at 1 week of pregnancy were protective factors against preterm birth, and high humidity (P95) and extremely high humidity (P99) at 4 weeks before delivery and at 1 week before delivery were risk factors for preterm birth. The effects were strongest at 4 weeks before delivery, with HRs of 1.257 (95% CI: 1.189–1.329) and 1.313 (95% CI: 1.228–1.404), respectively.

## 4. Discussion

### 4.1. Main findings

Our study found that an increased risk of preterm birth was significantly associated with high temperatures during pregnancy. Temperature during the early stage of pregnancy (1 and 4 weeks of pregnancy) was found to be associated with preterm birth in a U-shaped curve. The association between temperature and preterm birth was J-shaped during the preterm period (1 and 4 weeks before delivery). High temperatures (P95) and extremely high temperatures (P99) were both risk factors for preterm labor during all four exposure windows. Extremely low temperatures (P1) and low temperatures (P5) at 1 and 4 weeks of pregnancy were both risk factors for preterm birth and that extremely low temperatures (P1) and low temperatures (P5) at 1 and 4 weeks before delivery were protective factors for preterm birth. Low humidity was a protective factor for preterm birth, whereas high humidity was a risk factor for preterm birth. The correlation between relative humidity and risk of preterm birth was n-type at 1 week of pregnancy and the correlation between relative humidity and preterm birth at 1 and 4 weeks before delivery was J-shaped.

### 4.2. Strengths and limitations

Our study has several advantages. First, our study is the first study to explore the effects of both temperature and relative humidity during the early stage of pregnancy and before parturition on preterm birth in women of childbearing age. Second, our study is a long-term, population-based, large-sample cohort study on all women of childbearing age who participated in the NFPHEP and gave birth in Yunnan Province, resulting in high representativeness. In addition, we considered the effect of extreme weather, namely high temperatures, high humidity, low temperatures, and low humidity, on preterm birth to accurately describe the effects of meteorological factors on preterm birth.

Our study also has some limitations. The NFPHEP does not collect information regarding pregnancy complications such as gestational hypertension, gestational diabetes, and other factors affecting preterm birth (such as infection); this may have affected the results to a certain extent. Numerous studies have reported that air pollution is associated with premature birth, but we did not collect data on air pollution in Yunnan Province from 2010 to 2018. Subsequent studies should investigate air pollution and preventative measures in their analyzes.

### 4.3. Interpretation

Preterm birth is a global public health. The increases in global warming and extreme weather have received much attention in recent years, and many studies have been conducted on preterm birth that may be related to environmental factors. From a biological perspective, the early stage of pregnancy, specifically the weeks around conception when gametes mature, fertilization occurs, and the developing embryo forms, is a critical period (18). This stage is the most sensitive to environmental factors such as the availability of macronutrients and micronutrients and exposure to smoking, alcohol, drugs, and other teratogens (18). In addition, with the growth and development of the fetus, pregnant women’s physiology and metabolism undergo substantial adaptive changes that challenge their physical fitness (19). Studies have observed that exposure to social pressure (20), high temperature (21), pollutants (22), and radiation (23) before delivery in addition to depression (20) and anemia (24) in pregnant women may lead to adverse pregnancy outcomes.

Numerous studies have investigated the effect of temperature on preterm birth. An Australian study using Cox proportional hazards models and discovered that between July 1994 and December 2013, the relationship between temperature (mean, maximum, and minimum temperature) and risk of preterm birth formed a U-shaped curve. The HRs of preterm birth increased with temperature during the first two trimesters but nonsignificantly (5). A 2019 ecological study from the United States used a distributed lag nonlinear model and revealed a monotonic association between ambient temperature and risk of preterm birth. Days of extreme heat were associated with a higher relative risk of preterm birth, and days of extreme cold were associated with a slightly lower risk of preterm birth (17). A nationwide cohort study in China used the Cox proportional hazard regression models with random effect and discovered that the association between temperature and risk of preterm birth formed a U-shape curve during pregnancy (25). Relatively high temperatures during the first trimester were significantly associated with an increased risk of preterm birth [75th percentile vs. threshold: 1.78 (95% CI: 1.69–1.87); 95th percentile vs. threshold: 1.96 (95% CI: 1.85–2.08)]. Relatively high temperatures during the second trimester were significantly associated with an increased risk of preterm birth, and the effects tended to diminish but remained significant [75th percentile vs. threshold: 1.41 (95% CI: 1.34–1.47); 95th vs. threshold: 1.30 (95% CI: 1.23–1.38)]. Relatively low temperatures during the third trimester were significantly associated with an increased risk of preterm birth [25th percentile vs. threshold: 1.16 (95% CI: 1.13–1.20); 5th percentile vs. threshold: 1.17 (95% CI: 1.12–1.22)] (25). The results of a 2020 meta-analysis of 40 studies on the association between high temperatures and preterm birth revealed that the odds of a preterm birth increased 1.05-fold (95% CI: 1.03–1.07) per each 1°C increase in temperature. Most studies have reported a dose–response association where the rate of preterm birth increases with temperature and duration of heat exposure. Five studies on humid subtropical regions observed a U-shaped association between temperature and preterm birth, with the base covering the range of 18 to 25°C (6). Most studies show that high temperatures can increase the risk of preterm birth, but conclusions about the effect of low temperatures on preterm birth are inconsistent. Some studies showed the association between temperature and risk of preterm birth formed a U-shape curve, meaning that low temperatures also increased the risk of preterm birth, while other studies showed that low temperatures can reduce the risk of preterm birth. In this study, the effect of low temperatures on preterm birth varied with gestational age. Therefore, the conflicting results published to date might be due to difference in definitions of exposure, methods of exposure assessment, participants, gestational age and climate zones.

Few studies have investigated the effect of humidity on preterm birth. A Romanian study with a small sample analyzed the effects of atmospheric conditions on premature birth and discovered no significant correlation between humidity and preterm birth (26). A study in the Gambia identified July and October as two peaks in the incidence of preterm birth and an increased risk of preterm birth during the rainy season compared with the dry season (27). However, a study in Zimbabwe discovered that babies born early in the dry season were significantly more likely to be preterm than were those born late in the rainy season (28). In this study, the effect of humidity on preterm birth varied with gestational age. Studies have drawn differing conclusions, indicating the potential heterogeneity among the associations with region, climatic zone, population-based characteristics (e.g., socioeconomic conditions and adaptation), and other factors (17). In addition, the study in Romania had a small sample size, included pregnant women attending a single clinic, and used Pearson correlation and bivariate regression for correlation analysis (26). The study in Gambia involved all live births in 3 subsistence-farming villages of the West Kiang District in the Gambia, and used Fourier series and logistic regression for correlation analysis (27). The study in Zimbabwe involved pregnant women living in the residential area of Mbare in Harare, Zimbabwe, and used multiple linear and logistic regression for correlation analysis (28). Therefore, the sample size, the environment in which the study population lived, and statistical methods also influenced the association between meteorological factors and preterm birth.

Extreme weather has been documented to cause numerous health problems (4, 29, 30). A study in China observed that exposure to heat (>95th percentile) in hot areas increased the risk of preterm birth at the various stages of pregnancy more than did exposure to mild temperatures (5th–95th percentiles). The largest increase was during the 3 months before pregnancy [odds ratio (OR) = 1.229, 95% CI: 1.166–1.295]. Unlike heat exposure, cold exposure (<fifth percentile) in hot areas reduced the risk of preterm birth; the protective effect was strongest in the 3 months before pregnancy (OR = 0.784, 95% CI: 0.734–0.832). In mild and cold areas, cold exposure also reduced the risk of preterm birth. The effects of exposure to extreme ambient temperatures throughout pregnancy on preterm birth are similar to those of the aforementioned periods (1). A study in the United States observed that days of extreme heat and cold were associated with relative risks of preterm birth of 1.025 (95% CI: 1.015–1.036) and 0.985 (95% CI: 0.976–0.993), respectively. The association between extreme heat and risk of preterm birth varied by geographic region and climate zone, with stronger associations observed in the Southwest and Midwest United States and in the hot-dry–mixed-dry and cold–very cold climatic zones (17).

The exact mechanisms by which ambient temperatures cause preterm birth is unclear, but studies have proposed several hypotheses. First, dehydration and reduced blood flow caused by exposure to high temperatures may cause uterine contraction (31, 32). Second, exposure to high temperatures may increase cholesterol levels and blood viscosity, which may induce preterm delivery (31). In addition, existing evidence partially explains the association between exposure to low temperatures and preterm birth. First, exposure to low temperatures affects the circulatory system and causes an increase in blood viscosity and vasoconstriction, which in turn affects delivery (33). Exposure to cold temperatures exacerbates the risk factors for preterm delivery (pre-eclampsia and pregnancy-induced hypertension syndrome) because the cold environment leads to increased exposure to passive smoking and infection (15).

## 5. Conclusion

This study demonstrated that temperature and relative humidity during pregnancy were nonlinearly associated with risk of preterm birth. Temperature and relative humidity affect preterm birth differently for each pregnancy stage. High and low temperatures increase the risk of preterm birth during the early stage of pregnancy. High temperatures and high humidity increase the risk of preterm birth during the late stage of pregnancy. The meteorological factors on pregnancy outcomes such as premature birth should not be ignored.

## Data availability statement

The raw data supporting the conclusions of this article will be made available by the authors, without undue reservation.

## Ethics statement

The studies involving human participants were reviewed and approved by Institutional Review Board of the Chinese Association of Maternal and Child Health Studies (AMCHS-2014-6). The patients/participants provided their written informed consent to participate in this study.

## Author contributions

YW: writing–original draft, methodology, software, and data curation. JY: writing–original draft, methodology, and software. YY and CK: data curation and investigation. WJ and JL: writing–review and editing, supervision, and methodology. HY: data curation, investigation, project administration, and supervision. ML: conceptualization, funding acquisition, project administration, and supervision. All authors contributed to the article and approved the submitted version.

## Funding

This study was supported by the grant from National Natural Science Foundation of China (No. 71874003).

## Conflict of interest

The authors declare that the research was conducted in the absence of any commercial or financial relationships that could be construed as a potential conflict of interest.

The handling editor LP declared a shared affiliation with the authors at the time of review.

## Publisher’s note

All claims expressed in this article are solely those of the authors and do not necessarily represent those of their affiliated organizations, or those of the publisher, the editors and the reviewers. Any product that may be evaluated in this article, or claim that may be made by its manufacturer, is not guaranteed or endorsed by the publisher.
