# Supplementary material for: Effects of ambient temperature and relative humidity on preterm birth during early pregnancy and before parturition in China from 2010 to 2018: a population-based large-sample cohort study
Source: Front Public Health. 2023 Jun 20;11:1101283. doi: 10.3389/fpubh.2023.1101283 (PMC10319007; doi:10.3389/fpubh.2023.1101283)
Supplement: Supplementary file 1 [file Table_1.DOCX]

Table S1 Basic characteristics and the prevalence of preterm birth of participants

| **Basic characteristics** | **Total** | **%** | **Number of preterm births** | **Prevalence of preterm birth (%)** |
| --- | --- | --- | --- | --- |
| **Total** | 205771 | 100.00 | 18014 | 8.75 |
| **Age (years)** |  |  |  |  |
| ≤20 | 7541 | 3.66 | 931 | 12.35 |
| 21–25 | 74077 | 36.00 | 6608 | 8.92 |
| 26–30 | 80574 | 39.16 | 6604 | 8.20 |
| 31–35 | 30568 | 14.86 | 2570 | 8.41 |
| >35 | 12537 | 6.09 | 1221 | 9.74 |
| Missing | 474 | 0.23 |  |  |
| **Education** |  |  |  |  |
| Elementary school and below | 42220 | 20.52 | 4156 | 9.84 |
| Middle school | 110933 | 53.91 | 9738 | 8.78 |
| High school | 27931 | 13.57 | 2162 | 7.74 |
| College or higher | 21921 | 10.65 | 1631 | 7.44 |
| Missing | 2766 | 1.34 |  |  |
| **History of delivery** |  |  |  |  |
| No | 99783 | 48.49 | 8710 | 8.73 |
| Yes | 105988 | 51.51 | 9304 | 8.78 |
| **History of adverse pregnancy outcomes** |  |  |  |  |
| No | 149977 | 72.89 | 13227 | 8.82 |
| Yes | 55794 | 27.11 | 4787 | 8.58 |
| **BMI (kg/m^2^)** |  |  |  |  |
| <18.5 | 27229 | 13.23 | 2517 | 9.24 |
| 18.5-23.9 | 157935 | 76.75 | 13592 | 8.61 |
| 24.0-27.9 | 17831 | 8.67 | 1656 | 9.29 |
| ≥28.0 | 2432 | 1.18 | 212 | 8.72 |
| Missing | 344 | 0.17 |  |  |
| **Anemia** |  |  |  |  |
| No | 168587 | 81.93 | 14490 | 8.59 |
| Yes | 37003 | 17.98 | 3505 | 9.47 |
| Missing | 181 | 0.09 |  |  |
| **Smoking during pregnancy** |  |  |  |  |
| No | 200010 | 97.20 | 17361 | 8.68 |
| Yes | 3654 | 1.78 | 415 | 11.36 |
| Missing | 2107 | 1.02 |  |  |
| **Drinking during pregnancy** |  |  |  |  |
| No | 199333 | 96.87 | 17296 | 8.68 |
| Yes | 4224 | 2.05 | 471 | 11.15 |
| Missing | 2214 | 1.08 |  |  |
| **Fetal sex** |  |  |  |  |
| Female | 90178 | 43.82 | 7607 | 8.44 |
| Male | 97601 | 47.43 | 8786 | 9.00 |
| Missing | 17992 | 8.74 |  |  |

Table S2 Distribution of temperature and relative humidity by exposure window

| Meteorological factors | | 1 week of pregnancy | 4 weeks of pregnancy | 4 weeks before delivery | 1 week before delivery |
| --- | --- | --- | --- | --- | --- |
| Temperature  (℃) | Average | 16.08 | 16.09 | 15.99 | 16.03 |
|  | P1 (Extreme cold) | 2.64 | 4.29 | 1.99 | 3.52 |
|  | P5 (Cold) | 6.67 | 7.43 | 6.29 | 7.08 |
|  | P25 | 12.14 | 12.25 | 12.14 | 12.27 |
|  | P50 (Median) | 16.97 | 16.94 | 16.89 | 16.95 |
|  | P75 | 20.27 | 20.14 | 20.24 | 20.16 |
|  | P95 (Hot) | 23.26 | 22.92 | 23.09 | 22.79 |
|  | P99 (Extreme hot) | 25.26 | 25.00 | 25.04 | 24.84 |
| Relative humidity  (%) | Average | 71.91 | 71.82 | 73.05 | 73.04 |
|  | P1(Extreme dry) | 35.86 | 40.93 | 51.32 | 48.71 |
|  | P5(Dry) | 46.39 | 49.46 | 66.19 | 65.84 |
|  | P25 | 64.36 | 64.84 | 76.36 | 76.57 |
|  | P50 | 75.50 | 74.75 | 81.48 | 82.14 |
|  | P75 | 81.57 | 80.88 | 85.24 | 87.50 |
|  | P95(Wet) | 87.18 | 84.92 | 88.69 | 91.11 |
|  | P99(Extreme wet) | 91.00 | 88.82 | 43.15 | 38.43 |


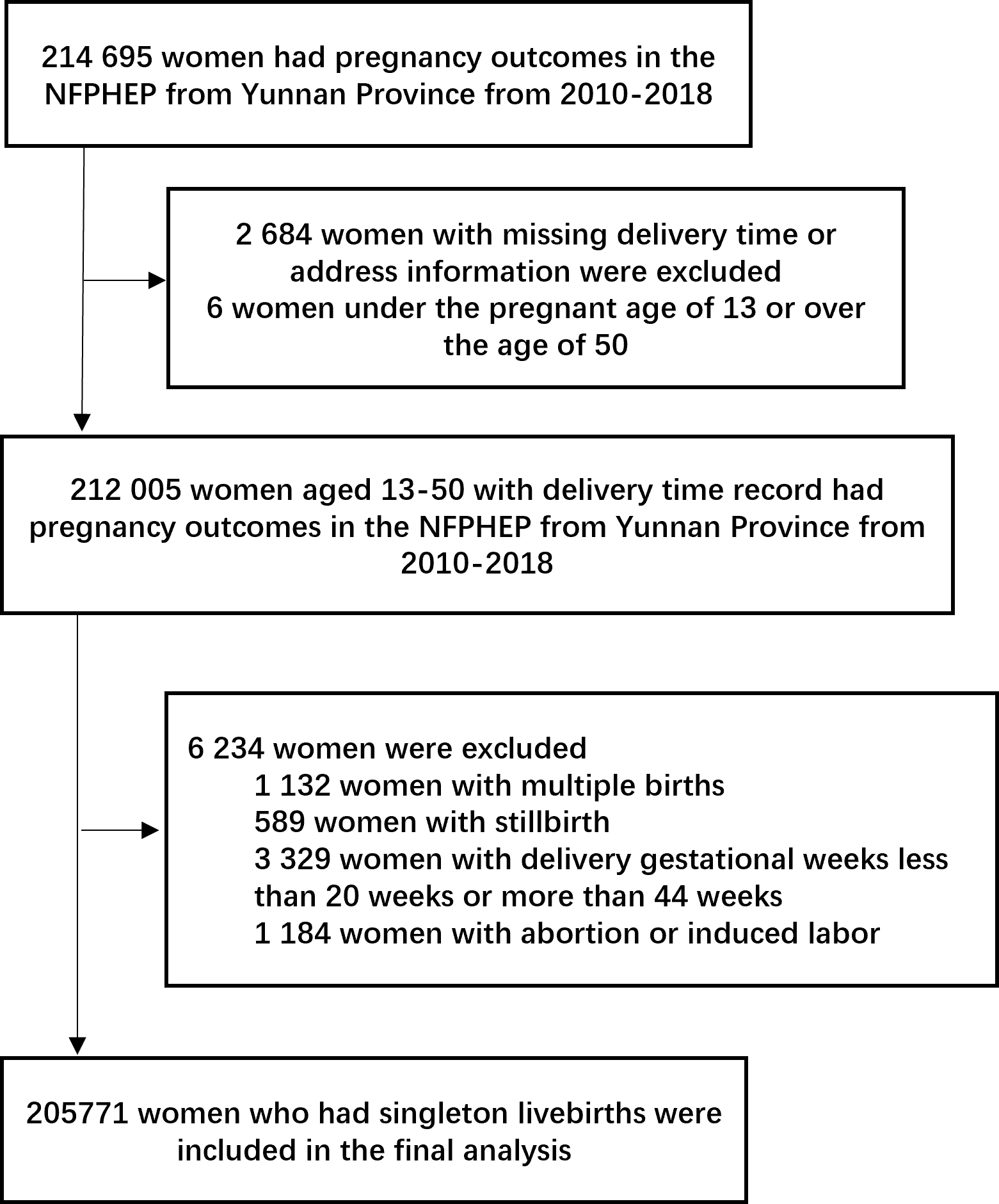


Figure S1 Inclusion and exclusion criteria of participants.


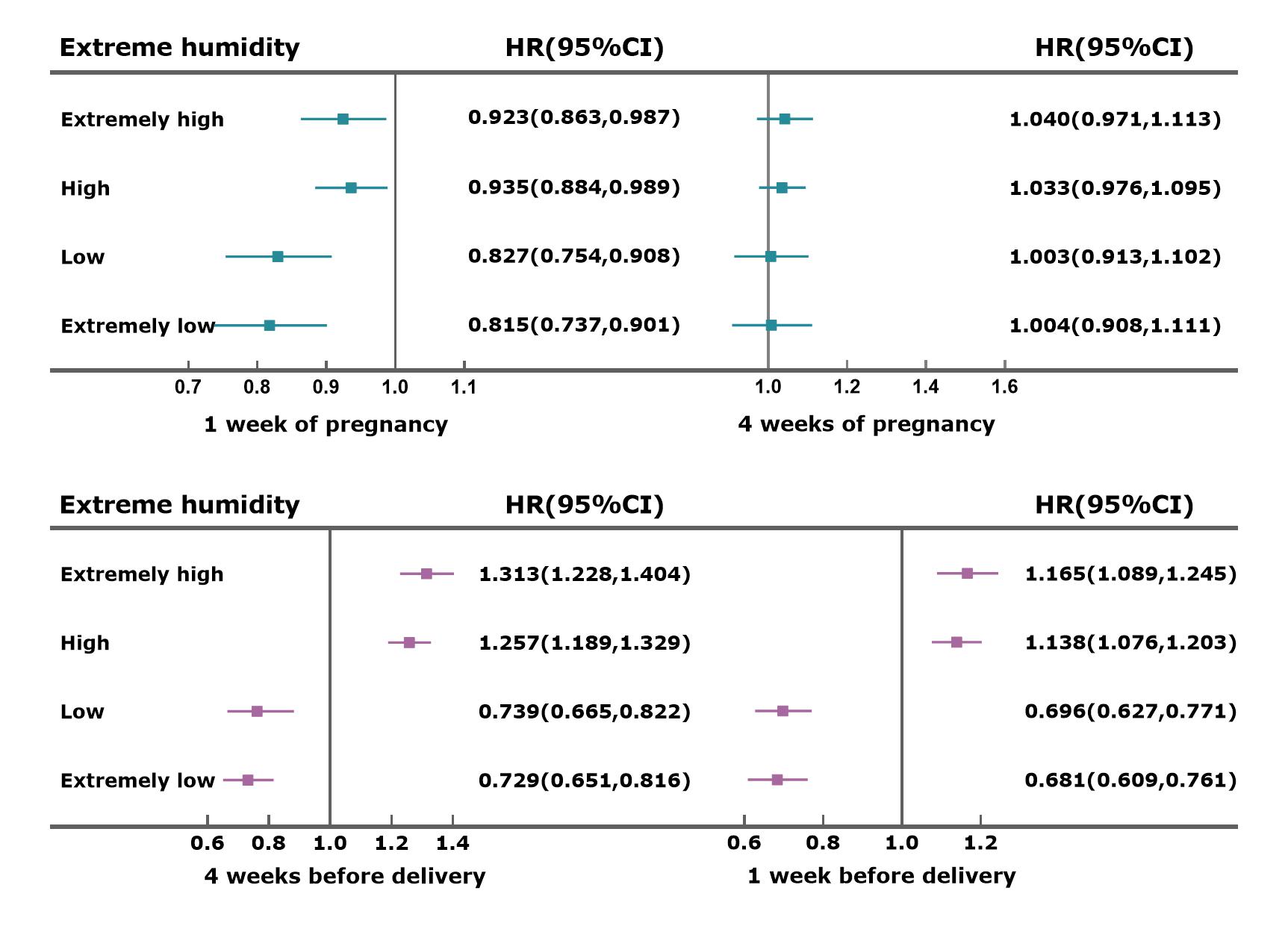


Figure S2 Effect of extreme humidity on preterm birth by exposure window
